# Cancer-Associated Fibroblast-Induced Resistance to Chemotherapy and Radiotherapy in Gastrointestinal Cancers

**DOI:** 10.3390/cancers13051172

**Published:** 2021-03-09

**Authors:** In-Hye Ham, Dagyeong Lee, Hoon Hur

**Affiliations:** 1 Department of Surgery, Ajou University School of Medicine, Suwon 16499, Korea; inhye@ajou.ac.kr (I.-H.H.); eay0401@ajou.ac.kr (D.L.); 2 Infamm-aging Translational Research Center, Ajou University School of Medicine, Suwon 16499, Korea; 3 Department of Biomedical Science, Graduate School of Ajou University, Suwon 16499, Korea

**Keywords:** cancer-associated fibroblasts, resistance, gastrointestinal cancer, chemotherapy, radiotherapy

## Abstract

**Simple Summary:**

Gastrointestinal (GI) cancers are primary malignant tumors associated with cancer-related deaths worldwide. Although chemotherapy and radiotherapy are essential modalities to improve patient survival, many patients show resistance to these therapies. Various clinical studies have suggested that cancer-associated fibroblasts (CAFs) play a significant role in this resistance. In this review, we discuss CAF-produced cytokines, chemokines, growth factors, and exosomes, as well as desmoplastic reactions, all of which could be involved in cancer therapy resistance. In the future, the heterogeneity of CAFs should be considered such that CAF subtypes involved in cancer therapy resistance may be identified, thus improving the efficacy of chemotherapy and radiotherapy in GI cancers.

**Abstract:**

In the past few decades, the role of cancer-associated fibroblasts (CAFs) in resistance to therapies for gastrointestinal (GI) cancers has emerged. Clinical studies focusing on GI cancers have revealed that the high expression of CAF-related molecules within tumors is significantly correlated with unfavorable therapeutic outcomes; however, the exact mechanisms whereby CAFs enhance resistance to chemotherapy and radiotherapy in GI cancers remain unclear. The cells of origin of CAFs in GI cancers include normal resident fibroblasts, mesenchymal stem cells, endothelial cells, pericytes, and even epithelial cells. CAFs accumulated within GI cancers produce cytokines, chemokines, and growth factors involved in resistance to therapies. CAF-derived exosomes can be engaged in stroma-related resistance to treatments, and several non-coding RNAs, such as miR-92a, miR-106b, CCAL, and H19, are present in CAF-derived exosomes and transferred to GI cancer cells. The CAF-induced desmoplastic reaction interferes with drug delivery to GI cancer cells, evoking resistance to chemotherapy. However, due to the heterogeneity of CAFs in GI cancers, identifying the exact mechanism underlying CAF-induced resistance may be difficult. Recent advancements in single-cell “omics” technologies could offer clues for revealing the specific subtypes and biomarkers related to resistance.

## 1. Introduction 

Cancers originating from the gastrointestinal (GI) tract, including the esophagus, stomach, colorectum, liver, and pancreas, are common malignancies and are the primary cause of cancer-related mortalities worldwide [1]. The core treatment strategy for GI cancers is surgical resection. However, patients with non-resectable or recurrent disease are predominantly treated with chemotherapeutic agents or radiation techniques as a palliative measure [2]. Targeting agents and immunotherapy are recently developed alternatives for improving the survival of GI cancer patients [3]. However, most patients with advanced-stage GI cancers are resistant to these treatment modalities; thus, their survival rates remain dismal.

Several studies have investigated the mechanisms underlying resistance to therapy in cancers originating from the GI tract. These studies have focused on the tumor cells themselves, such as drug efflux through transmembrane transport proteins and anti-apoptotic protein activation [4,5]. However, to date, agents that block these pathways have not yet been applied in clinical settings. Moreover, numerous studies have revealed that the tumor microenvironment (TME) may play a pivotal role in resistance to chemotherapy and radiotherapy [6,7]. The TME of solid cancers comprises various non-cancerous cells, the extracellular matrix, and soluble factors [8,9] that enhance tumorigenesis, invasion, metastasis, and therapy resistance in cancer cells. Therefore, targeting agents that block the interaction between cancer cells and the TME may improve treatment outcomes in GI cancer patients [10]. 

Cancer-associated fibroblasts (CAFs) constitute a significant component of the TME in GI cancers. They are involved in cancer invasion and tumor growth through their interaction with cancer cells and immune microenvironments [11,12]. Numerous studies have reported that CAFs can trigger the resistance of cancer cells to treatments [13,14,15,16]. Therefore, CAFs have emerged as a novel treatment target to improve the efficacy of chemotherapy and radiotherapy in GI cancers. However, drugs targeting CAFs have not yet been administered to patients. 

Herein, we introduce clinical evidence for CAF-induced resistance to treatments and describe the activity of CAFs in GI cancers. Furthermore, we summarize current research regarding the possible mechanism through which CAFs may evoke resistance to chemoradiotherapy in GI cancers.

## 2. Clinical Evidence for the Role of CAFs in Chemotherapy and Radiotherapy Resistance in GI Cancer

The desmoplastic reaction developed by the recruited fibroblasts is prominently observed in progressed GI cancers [17], and this reaction has been considered a major cause of resistance to chemoradiotherapy [18]. Some clinical studies have demonstrated that high desmoplasia is significantly correlated with poor clinical outcomes in patients with GI cancers, such as pancreatic ductal adenocarcinoma (PDAC) and colorectal cancer (CRC) [19,20,21]. Therefore, treatment strategies targeting tumor desmoplasia have mainly tried to improve the survival of patients with advanced GI cancers [11]. For example, the monoclonal antibody for fibroblast activation protein (anti-FAP mAb) showed some therapeutic effects in CRC without severe toxicity in the early phase of a clinical trial [22]. Additionally, recent phase II clinical trials testing angiotensin I receptor blockers as inhibitors of CAF activation and pegvorhyaluronidase alfa as a decomposer of hyaluronan accumulated by CAFs; these trials have described improved outcomes in PDAC patients [23,24]. Although these agents have not yet been approved as a treatment of choice for GI cancers, accumulating evidence suggests that targeting CAFs in GI cancers is promising (Table 1).

It has been confirmed through immunohistochemistry (IHC) that CAF accumulation in GI cancers is related to chemotherapy resistance. Ma et al. performed IHC for alpha-smooth muscle actin (α-SMA) in paraffin-embedded formalin-fixed (PEFF) tissues of gastric cancer (GC) patients treated with chemotherapy. The results showed that the GC tissues of patients showing resistance to chemotherapy contained more α-SMA-positive CAFs than the chemosensitive patients [25]. Other CRC studies also reported a significant correlation between a high proportion of α-SMA-expressing CAFs and resistance to 5-fluorouracil plus oxaliplatin-based chemotherapy [26]. 

The expression of CAF-derived molecules in human GI cancer tissues could be investigated to provide clinical evidence. Some researchers have reported a direct correlation between biomarkers originating in stromal cells and response to neoadjuvant treatment. The expression of the two markers FAP-α and C-X-C motif chemokine ligand (CXCL) 12, known as stromal cell-derived factor 1 (SDF-1), was positively associated with poor clinical outcomes in rectal cancer patients who underwent neoadjuvant chemoradiation [27,28]. Although chemoradiotherapy is the most popular modality for esophageal cancers (ESOCs), patients have frequently exhibited resistance to therapies, resulting in poor outcomes [32]. One study described the expression of CXCL1 in ESOC tissue specimens biopsied after chemoradiation. We concluded that the upregulation of CXCL1 in CAFs was an independent prognostic factor in these patients [29]. In addition, positive transforming growth factor-beta (TGF-β) expression in CAFs of ESOC tissues was significantly correlated with poor survival outcomes in patients treated with chemoradiotherapy [30]. Another group reported that high PAI-1 expression in CAFs led to considerably worse progression-free survival in ESOC patients treated with cisplatin [31].

**Figure 1:**
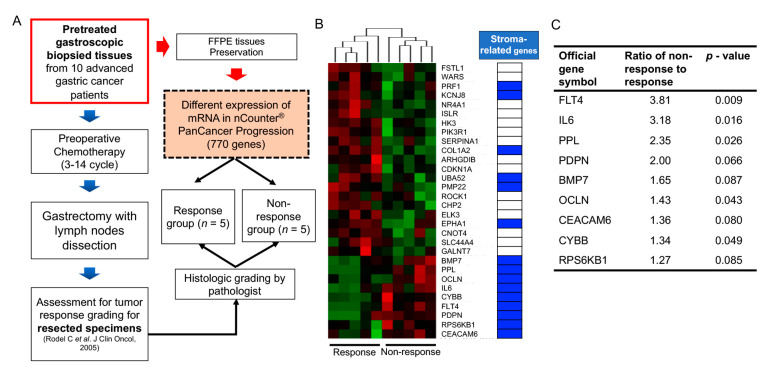
Gene expression patterns in pretreated gastroscopically biopsied tissues of patients who underwent preoperative chemotherapy (modified from Ham et al., 2019, Mol Cancer [15]. (**A**). Flow diagram presenting the study scheme for the comparison of gene expression patterns using the nCounter system between chemotherapy responders and non-responders. (**B**). Heatmap depicting different gene expression patterns between chemotherapy responders and non-responders. Blue-colored cells in the table to the right of the heatmap indicate stroma-related genes. (**C**). Nine stroma-related genes were found in non-responders.

Large-scale cancer genome studies using high-throughput technologies have provided comprehensive molecular profiling information for solid cancers [33]. The Cancer Genome Atlas (TCGA) consortium has suggested molecular subgroups and treatment targets based on a genome-scale analysis using bulk tumors of large cohorts [34,35,36,37,38]. However, considering the role of non-cancerous cells in the bulk tumors on cancer progression and therapeutic efficacy, the meanings of these cell fractions should be investigated. Algorithms including ESTIMATE [39], CIBERSORT [40], EPIC [41], and MCP-counter [42] can predict the proportion of stromal or immune cells in bulk cancer tissues. Consequently, the implications of the accumulation of these cells in GI cancer patient prognosis can be inferred. Recent high-throughput transcriptome analyses of GI cancers have highlighted that stroma-related genes have unfavorable outcomes in patients with various types of GI cancers, including GC, CRC, PDAC, and hepatocellular carcinoma [43,44,45,46,47]. However, these results were obtained using surgical specimens from patients who underwent curative resection, with or without subsequent adjuvant systemic treatment. To define the correlation between gene expression and response to chemotherapy, expression analyses in pretreated samples from patients subjected to preoperative chemotherapy can undoubtedly reflect their responsiveness to chemotherapy based on gene expression. Our recent data obtained using NanoString transcriptome analysis revealed that stroma-related gene expression in pretreated endoscopic biopsy tissues of GC patients who underwent preoperative chemotherapy significantly correlated with an inadequate response to chemotherapy [15]. Although NanoString transcriptome analysis screens a limited number of genes, it could be applied to a small number of samples, such as endoscopic biopsy specimens. The results implied that the high expression of stroma-related genes in the biopsied tissues of GC patients might require a novel treatment strategy. This strategy may improve the efficacy of chemotherapy for patients with GC (Figure 1). However, our study had some limitations, including a low number of enrolled patients. Another study with a large number of GC patients who had undergone neoadjuvant treatment showed that several genetic mutations could serve as predictive markers for chemotherapy response [48]. However, future studies investigating TMEs should be conducted to assess their role in therapy resistance. 

Collectively, these findings indicate that CAF accumulation or CAF-specific markers in malignant tumors originating from the GI tract are significantly related to chemotherapy or radiotherapy resistance. Therefore, the mechanisms underlying the interaction between CAFs and cancer cells would act as excellent targets to improve the responsiveness of GI cancer patients to chemoradiotherapy.

## 3. Origin of CAFs in GI Cancer

**Figure 2:**
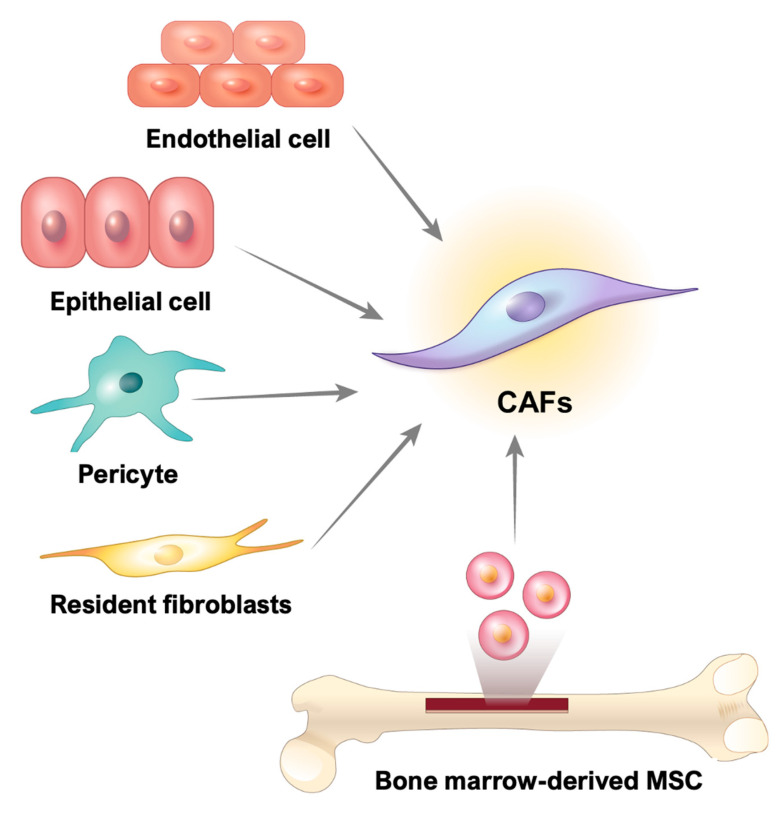
Origins of cancer-associated fibroblasts (CAFs). Sources of CAFs in gastrointestinal cancers include resident fibroblasts, endothelial cells, epithelial cells, pericytes, and bone marrow-derived mesenchymal stem cells. CAFs: cancer-associated fibroblasts, MSC: mesenchymal stem cell.

CAFs are fibrotic cells involved in tumor malignancy; however, the origin of these cells in GI cancer remains unclear. Numerous studies have reported that CAFs may be derived from resident fibroblasts, smooth muscle cells, endothelial cells, pericytes, bone marrow-derived stem cells, and even epithelial cells [49,50] (Figure 2).

Genetic and functional comparisons between fibroblasts isolated from surgically resected cancers and paired healthy tissues are relatively easier to perform than those isolated from other cell types; therefore, resident fibroblasts have been extensively explored in this context [51]. The unique characteristics of CAFs compared to normal resident fibroblasts could indicate the potential mechanism underlying the transdifferentiation of normal fibroblasts to CAFs in GI cancers. TGF-β is produced by colon cancer cells and activates the differentiation of residual colon fibroblasts into CAFs during colon cancer progression. These activated CAFs upregulate the expression of activated markers, such as α-SMA and FAPs, and produce large amounts of glycoproteins, including tenascin-C and collagen maturation enzymes, for extracellular matrix (ECM) remodeling [52]. GC cells of the scirrhous subtype also produce TGF-β, which indicates the expression of α-SMA in normal residual fibroblasts [53]. Moreover, the aforementioned study proposed that TGF-β could be reciprocally involved in the CAF-induced stemness of scirrhous GC cells and demonstrated that anti-TGF-β antibody had an inhibitory effect on tumor growth. 

In PDAC, one of the distinct origins of CAFs may be pancreatic stellate cells (PSCs), the resident mesenchymal cells of the noncancerous pancreas [54]. Similar to GC, PDAC cell-induced TGF-β can activate PSCs and increase the production of ECM components such as fibronectin, collagen, and tenascin-C [55]. Additionally, the sonic hedgehog (SHH) protein expressed in PDAC cells contributes to tumor progression via the differentiation and motility of PSCs or resident fibroblasts that already exist in the pancreatic tissue [56]. However, despite the positive effects of an antibody against SHH in the PDAC preclinical animal model [56], a clinical trial showed that the SHH inhibitor did not synergize PDAC patients with gemcitabine treatment [57].

Bone marrow-derived mesenchymal stem cells (MSCs) may act as a potential source of CAFs in inflammation-induced GC [58]. The *Helicobacter*-induced GC mouse model reveals that CAFs are derived from α-SMA-positive myofibroblasts in the bone marrow, and these CAFs can form a tumor niche in the gastric wall and undergo tumor progression. The MSCs recruited from the bone marrow may act as a source of CAFs in PDAC and pancreatic endocrine tumors [54,59]. MSCs exposed to PDAC cells are activated into CAF-secreting tumor-promoting proteins such as hepatocyte growth factor (HGF), epidermal growth factor (EGF), and interleukin-6 (IL-6). These proteins stimulate microvascularization, changes in the composition of the stromal framework, and tumor growth through the paracrine system [54].

Other noncancerous cells, such as endothelial cells, pericytes, and even epithelial cells, which accumulate in GI cancer, can be transformed into CAFs through cell transition mechanisms. A study using a pancreatic islet tumor mouse model revealed that fibroblast-specific protein 1 (FSP1) and CD31 double-positive cells exist in the TME. Previous studies have reported that TGF-β mediates the transition from endothelial cells to mesenchymal cells in cardiac tissues [60]. Since abundant TGF-β expression was also apparent in this tumor, the authors suggested that TGF-β-exposed pancreatic endothelial cells could be a source of CAFs [61]. 

Vascular pericytes are multifunctional mural cells that surround endothelial cells [62], and they are crucial in the neoangiogenesis and survival of endothelial cells during tumorigenesis [63]. Emerging evidence has indicated that neural/glial antigen 2 (NG2)-expressing pericytes are transformed into CAFs through platelet-derived growth factor-BB (PDGF-BB) stimulation in a CRC xenograft model [64]. Moreover, the expression of *PDGFB* and *FSP1* in various types of solid tumors, including CRC, is significantly correlated with poor patient prognosis [64].

Furthermore, epithelial cells of GI organs could be a source of CAFs during carcinogenesis. In genetic PDAC mouse models, pancreatic epithelial cells are transformed into mesenchymal cells through epithelial–mesenchymal transition; these cells have a fibroblast-like phenotype, express FSP1, and are deeply involved in tumor formation. However, although these FSP1-expressing cells are similar to CAFs, it is still unclear whether these cells could be a significant source of CAFs in PDAC tumors [65]. Therefore, further studies are required to verify whether epithelial cells are a crucial source of CAFs in GI cancers.

## 4. Factors Related to CAF-induced Resistance to Cancer Treatment

### 4.1. Cytokines and Chemokines 

Cytokines and chemokines are inflammatory mediators secreted by cancer cells or tumor stromal cells in the TME. They can stimulate tumor-promoting pathways, including proliferation, metastasis, and progression in an autocrine or paracrine manner [66]. Moreover, the cytokines and chemokines in the TME are deeply related to chemoresistance and poor prognosis in cancer patients [67,68]. The CAFs in GI cancers could act as a source of various TME cytokines and chemokines. 

IL-6, a multifaceted cytokine related to infection or injury response, plays a predominant role in cancer progression [69]. Recent studies have suggested that IL-6 is mainly secreted by CAFs, and CAF-derived IL-6 can induce an inadequate response to chemotherapy in GI cancers, including CRC, esophageal cancer (ESOC), and GC [15,70,71,72]. Qiao et al. [70] demonstrated that IL-6 from CAFs contributes to chemoresistance by activating the signal transducer and activator of transcription 3 (STAT3)/nuclear factor-κB (NF-κB) pathway and subsequently upregulating C-X-C motif chemokine receptor (CXCR) 7 expression in ESOC cells. Additionally, other studies demonstrated that stromal IL-6 increases the expression of cancer stem cell (CSC) markers and consequently induces resistance to chemoradiotherapy in ESOC patients [72]. Moreover, we demonstrated that CAF-derived IL-6 stimulates the Janus kinase 1 (JAK1)/STAT3 pathway in GC cells in a paracrine manner [15]. Furthermore, in human GC tissues, high expression of stroma-related genes, including *IL-6*, is significantly correlated with resistance to chemotherapy. Eventually, we found that the IL-6 receptor monoclonal antibody, tocilizumab, rescued CAF-induced resistance to chemotherapy in various experimental models [15]. Taken together, these studies show that CAFs may act as a source of IL-6 in the GI cancer microenvironment; as such, IL-6 inhibition could be a novel therapeutic strategy to decrease CAF-induced resistance to cancer therapies.

The human chemokine CXCL1, termed the GRO-1 oncogene, specifically binds to CXCR2, a member of the G-protein-coupled receptor family [73]. Zhang et al. [29] reported that the expression of CXCL1 in CAFs isolated from ESOC tissues was higher than that in normal fibroblasts. CAF-derived CXCL1 could be involved in tumor radiotherapy resistance by activating the MEK/ERK pathway.

CXCL12, also known as SDF-1, is mainly secreted from the stromal cells of solid tumors and is a primary ligand of the membrane receptor CXCR4 [74]. The role of CXCL12 has been explored in PDAC; several studies have reported that PSCs secrete CXCL12 into the TME, which promotes the resistance of PDAC cells to chemotherapy in a paracrine manner [75,76,77]. Secreted CXCL12 may activate the FAK/ERK1/2/AKT signaling pathways in PDAC cells, thereby inducing resistance to gemcitabine [75,77]. CXCL12-induced activation of this signaling pathway increases the transcriptional activities of β-catenin and NF-κB, thus leading to an elevated expression of survival proteins such as Bcl-2 [77]. Moreover, these CXCL12-activated pathways increased the secretion of IL-6 in PDAC cells related to chemoresistance [75]. Therefore, the small-molecule CXCR4 antagonist plerixafor has been used to abolish CXCL12-induced PDAC growth and chemoresistance [75,77]. A recent clinical trial demonstrated that the combination of plerixafor and chemotherapy increased the response rate of conventional chemotherapy in a hematological malignancy [78]; hence, this combination may be an effective chemosensitizer for GI cancer. Radiotherapy has been perioperatively administered to patients with PDAC. PDAC patients who undergo curative resection can be treated with radiotherapy to suppress cancer recurrence. Radiotherapy for inoperable PDAC patients can be used for symptom palliation [79]. One study concluded that CAF-derived CXCL12 promotes PDAC cell resistance to radiotherapy through CXCR4 activation [80]. This result suggests that CAF-induced CXCL12/CXCR4 signaling could be a novel therapeutic target to improve the effectiveness of radiation [80]. 

Chemotherapeutic agents can stimulate the production of various secretory proteins in CAFs. In experimental models of CRC, chemotherapy-stimulated CAFs enhance the secretion of specific cytokines such as IL-17A, and increased serum levels of IL-17 have been observed in CRC patients with chemoresistance. CAF-secreted IL-17A promotes chemoresistance in cancer-initiating cells (CICs) through the NF-κB pathway and increases CIC self-renewal, invasion, and tumor growth in vivo [81].

### 4.2. Growth Factors 

Cancer cells usually express various receptor tyrosine kinases (RTKs) that can mediate downstream signaling pathways, such as mitogen-activated protein kinase (MAPK) and phosphatidylinositol-3-OH kinase, which can contribute to therapy resistance [82,83]. Although RTKs are highly activated through genetic mutations in various cancers, growth factor stimulation is a crucial mechanism for RTK-induced inadequate therapeutic responses [84]. In particular, if growth factors are secreted from CAFs, they can act as messengers for cell–cell communication.

In addition, cancer-secreted TGF-β can enhance the transition of resident fibroblasts into CAFs, as mentioned in Section 3, and CAF-secreted TGF-β is involved in cancer therapy resistance in GI cancer cells. In ESOC, CAF-conditioned media includes a higher concentration of TGF-β1 than the conditioned media from normal fibroblasts [30]. Consequently, CAF-derived TGF-β1 enhances resistance to cisplatin and taxol, and TGF-β1 expression in CAFs is significantly related to poor prognosis in ESOC patients subjected to chemoradiotherapy [30]. Another study showed that miR-27a/b converts normal fibroblasts to CAFs in ESOC, and the converted CAFs enhance resistance to cisplatin by secreting TGF-β1 [85]. Both studies demonstrated that the TGF-β1 inhibitor LY2157299 could improve the response of ESOC cells to various chemotherapeutic agents both in vivo and in vitro. 

The insulin-like growth factor (IGF) family plays a crucial role in regulating cell proliferation and apoptosis by activating transmembrane receptors; thus, it contributes to resistance to GI cancer therapies [86]. Ireland et al. [87] suggested that CAFs could be a source of IGF-1 and IGF-2 in PDAC and consequently activate insulin/IGF receptors on PDAC cells. They also demonstrated that the inhibition of IGFs sensitizes PDAC cells to gemcitabine. The mechanism underlying the upregulation of IGF-1 expression in PDAC CAFs was evaluated by Xiao et al., [88] who demonstrated that the PDAC-enhanced methylation of suppressor of cytokine signaling 1 (SOCS1) plays a pivotal role in the transition of normal fibroblasts to CAFs. In turn, SOCS1 downregulation was associated with IGF-1 expression in CAFs. Moreover, radiotherapy may trigger the secretion of IGF1, which could be involved in the resistance of rectal cancer to radiotherapy. Radiation-activated CAFs promote CRC cell survival by activating the IGF-1 receptor; thereafter, the neutralization of this receptor in a CRC cancer animal model reduces metastasis [89]. 

HGF is a major secretory protein of CAFs in solid tumors that promotes cancer cell survival and provides therapeutic resistance [90]. CAF-secreted HGF increases the proportion of tumor-initiating cells of HCC through c-MET activation. Activated c-MET in tumor-initiating cells further activates the ERK/FRA1/HEY1 cascade, which is related to chemotherapy resistance [91].

Cetuximab, an EGF receptor (EGFR) monoclonal antibody, is a molecular targeted agent that improves the survival of patients with CRC without *Kras* mutation [92]. Luraghi et al. [93] reported that CAF-secreted HGF activates resistance to EGFR inhibitors in experimental models. CAF-induced HGF could also play a pivotal role in radiotherapy resistance. One study demonstrated that the levels of secreted HGF in irradiated fibroblasts isolated from ESOC were higher than those in non-irradiated controls. HGF derived from irradiated fibroblasts increases wound healing, migration, and invasion [94]. 

### 4.3. Exosomes

Various studies have examined the role of exosomes in cancer progression. The exosome, a microvesicle of endocytic origin with a diameter of 30–150 nm, is secreted by many cells. As exosomes comprise a lipid bilayer containing various bioactive molecules, such as DNA, microRNAs (miRNAs), proteins, long non-coding RNAs (lncRNAs), circular RNAs, and lipids, they function as natural vehicles in cell–cell communication by transferring genetic messages. Exosomes secreted from various cells within tumors enable communication among tumor cells surrounding the TME and in distant organs or tissues, leading to the promotion of metastasis and therapy resistance [95]. Therefore, exosomes generated from CAFs would be suitable messengers to enhance the resistance of GI cancer cells to therapy.

The function of CAF-derived exosomes in cancer therapy resistance was initially investigated in CRC. Hu et al. [96] reported that CAF-derived exosomes promote drug resistance by mediating the activation of the Wnt signaling pathway in CSCs in CRC. Next, the kinds of elements included in CAF-derived exosomes that can enhance resistance to therapies have recently been evaluated. Non-coding RNAs (ncRNAs) such as miRNAs and lncRNAs have been suggested as crucial molecules for exosome-mediated communication between CAFs and GI cancer cells [97]. miRNAs are a subtype of ncRNAs that contain 17–25 bp of ncRNA and usually suppress messenger RNA translation by targeting the 3′-UTR; thus, miRNAs facilitate the epigenetic regulation of gene expression and can control the pathological process in cancers [98]. Notably, several miRNAs present in exosomes are involved in drug resistance in cancers [99]. Regarding GI cancers, CAFs of CRC secrete miR-92a-3p-enriched exosomes into the TME. When exosomal miR-92a-3p is transferred to CRC cells, it promotes migration, invasion, metastasis, stemness, and drug resistance. Thus, blocking the function of exosomal miR-92a-3p secreted by CAFs could be used as an alternative modality for therapy resistance in CRC [100]. In GC, Zhang et al. [101] demonstrated that CAF-secreted exosomal miR-522 regulates arachidonate lipoxygenase 15 (ALOX15) expression and is closely related to lipid reactive oxygen species (ROS) production. They suggested that blocking lipid-ROS production might be a novel mechanism for acquired drug resistance. Thus, targeting exosomal miR-522 could be a modality to increase the sensitivity of GC patients to chemotherapy [101]. Furthermore, when miR-106b in the PDAC CAF-derived exosomes is transferred into PDAC cells, it mediates resistance to gemcitabine [102]. Therefore, detecting miRNAs in CAF-derived exosomes of GI cancer could provide efficient biomarkers to predict chemotherapy response. Moreover, targeting the function of these miRNAs could serve as a promising tool for improving the drug response in GI cancers.

Exosomes can also contain lncRNAs, which are nucleotide transcripts over 200 bp in length that are not translated into proteins [103]. The function of lncRNAs in the resistance of various cancers to therapies has recently been proposed; this could be the central mechanism related to CAF exosome-derived drug resistance in GI cancer. Deng et al. [104] demonstrated that CAF-derived exosomes express CRC-associated lncRNA (CCAL) more highly than normal fibroblasts, and these CCAL-enriched exosomes may drive cancer cells to oxaliplatin resistance. In addition, the transfer of CCAL could function as an oncogenic lncRNA and induce Wnt/β-catenin pathway activation in CRC cells. Therefore, CCAL may represent a biomarker and druggable target for CRC chemoresistance. Another CAF-derived exosomal lncRNA, H19, also promotes stemness and chemoresistance in CRC cells [105]. Transferred H19 could activate the β-catenin pathway in CRC cells by blocking the function of miR-141, which could inhibit stemness. 

Furthermore, CAF-derived exosomes may also be associated with resistance to radiotherapy. Liu et al. [106] reported that CAF-derived exosomes confer robust radiation resistance in CRC cells by activating the TGF-β signaling pathway.

### 4.4. Other Mechanisms 

The other secretory materials produced from CAFs and CAF-induced intratumoral pressure escalation could be involved in the resistance to GI cancer therapies. 

Plasminogen activator inhibitor-1 (PAI-1) is a secreted protein that not only enhances angiogenesis, but also promotes the invasion and metastasis of certain cancer cells [107,108]. PAI-1 is secreted from CAFs, and one study reported that cisplatin-treated CAFs increase PAI-1 secretion [31]. CAF-secreted PAI-1 enhances progression and chemoresistance by activating the AKT/ERK1/2 signaling pathway and inhibiting caspase-3 activity and ROS accumulation in ESOC cells. Moreover, the high expression of PAI-1 in CAFs is correlated with poor prognosis in ESOC patients; consistently, the PAI-1 inhibitor tiplaxtinin presents synergistic effects with cisplatin both in vitro and in vivo [31].

CAF-secreted perlecan (heparin sulfate proteoglycan 2, HSPG2) plays a critical role in resistance to chemotherapy in PDAC. CAFs isolated from genetic PDAC mouse models were reprogrammed in mouse PDAC cells with a *P53* mutation. The reprogrammed CAFs increased the stromal deposition of HSPG2, which created a prometastatic and chemoresistant environment in pancreatic cancer cells [109]. Another CAF-induced secreted protein that protects PDAC cells from gemcitabine is laminin A1. Although PDAC cells secrete transglutaminase, they do not increase the cytotoxicity of gemcitabine directly; however, transglutaminase enhances the secretion of laminin A1 from CAFs, and secreted laminin A1 secreted in the TME could protect PDAC cells from chemotherapeutic agents such as gemcitabine [110]. 

CAF-secreted T-lymphoma invasion and metastasis-inducing protein-1 (TIAM1) is a key regulator of chemoresistance in CRC cells [111]. CAF-derived conditioned media increased resistance to chemotherapy through TIAM1 overexpression, and TIAM1-associated drug sensitivity was validated using a xenograft mouse model.

CAFs are the center of desmoplastic reactions in GI cancer as well as a source of secreted proteins; thus, they interfere with drug delivery by collapsing the peritumoral capillaries and increasing intratumoral interstitial pressure. This concept has been suitably evaluated in a mouse model of PDAC, wherein GI tumors exhibit profuse desmoplastic reactions. The accumulation of hyaluronic acid (HA) produced from CAFs during PDAC progression was found to be responsible for enhancing intratumoral pressure, thus acting as a barrier for drug diffusion in the mouse model. Provenzano et al. [112] suggested that enzymatic dissolution of stromal HA could increase the efficacy of cancer drugs by remodeling PDAC stromal lesions.

## 5. Heterogeneity of CAFs in GI Cancers

Tumor heterogeneity has recently been considered a crucial factor underlying resistance to antitumor therapies, including both non-cancerous stromal cells and cancer cells. In addition, various subtypes of CAFs exist [113,114,115]. Therefore, clarifying the mechanism underlying CAF heterogeneity may provide crucial information on GI cancer progression and would enable the development of novel therapeutic approaches.

Of all the GI cancers, CAF heterogeneity is best understood in PDAC. Ohlund et al. [116] reported the existence of distinct subtypes of CAFs based on their localization within the primary tumor. The α-SMA^high^ CAF subtype is in direct contact with cancer cells, whereas α-SMA^low^ CAFs are located distally from cancer cells, releasing proinflammatory cytokines [116]. Other studies have also explored the function of α-SMA^high^ CAF subtypes. Genetically engineered PDAC mouse models with α-SMA-negative fibroblasts result in more aggressive tumors and gemcitabine resistance [117,118]. Presumably, α-SMA-expressing CAFs may suppress tumor immunity and increase tumor vascularization. 

These findings suggest that the CAF subtype can be characterized and identifying the specific subtypes of CAFs that play a crucial role in GI cancer progression could present novel targets for therapy. The recent development of single-cell transcriptome technology for solid tumors has shed light on the composition of various cancerous and non-cancerous tissues, as well as the heterogeneous population of accumulated cells, through gene expression patterns [119,120]. Elyada et al. [121] conducted a single-cell analysis of PDAC CAFs and found the following three subtypes: myofibroblastic, inflammatory, and antigen-presenting. Although they did not demonstrate the function of these subtypes in chemoradiotherapy resistance, this advanced technology can provide detailed information regarding CAF heterogeneity in GI cancers. 

## 6. Conclusions and Future Perspectives

**Figure 3:**
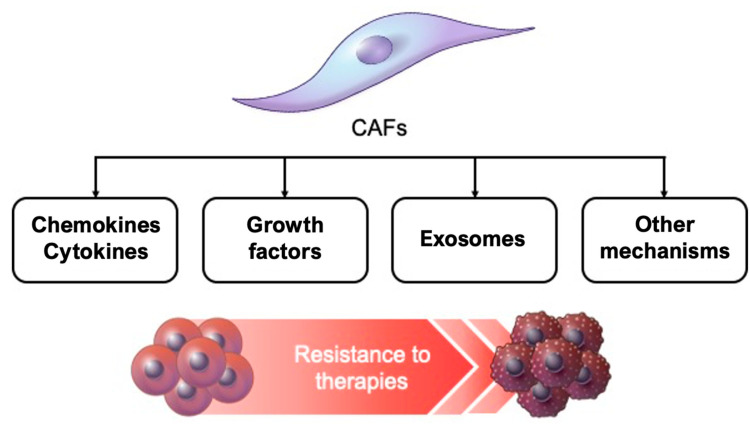
Cancer-associated fibroblasts (CAFs) orchestrate the resistance to chemoradiotherapies in the tumor microenvironment. CAFs secrete abundant chemokines, cytokines, growth factors, exosomes, and other factors.

The role of CAFs in GI cancer progression has been explored extensively over the past decade [122,123]. However, in the current review, we have focused on a substantial amount of evidence related to the correlation between CAFs and chemotherapy and radiotherapy resistance in GI cancers (Figure 3, Table 2). CAFs accumulated in GI cancers secrete IL-6 or CXLC12, which can activate signal transduction with respect to drug resistance. Inhibitors of IL-6 and CXCL12, such as tocilizumab and plerixafor, respectively, exert chemosensitizing effects on GI cancers. Growth factors, such as TGF-β1, are crucial in CAF-induced resistance to therapies; however, therapeutic strategies to target CAFs for GI cancer treatment have not yet been applied in clinical settings. More complicated mechanisms may be involved in the communication between CAFs and GI cancer cells. Recent studies have demonstrated that small extracellular vesicles, such as exosomes containing miRNAs and lncRNAs, can control the epigenetic regulation of genes related to drug response. Nevertheless, exosome-based controls for improving therapeutic responses remain underdeveloped. Another factor that complicates this avenue of research is the heterogeneity of CAFs in GI cancer. Heterogeneous CAF populations must be precisely defined to determine the specific subtypes related to therapy resistance, but this remains a challenge. Recent advances in technologies, such as single-cell “omics,” will aid the exploration of CAF subpopulations and novel biomarkers related to chemotherapy and radiotherapy resistance in GI cancers.

## Figures and Tables

**Table 1:** Clinical studies investigating the role of CAFs in resistance to chemotherapy and radiation therapy in gastrointestinal cancers.

Patients	Methods	Marker	Resistance to	Results	References
30 pts with gastric cancer	IHC	α-SMA	Chemotherapy	High expression in the non-response group	[25]
71 pts with colorectal cancer	IHC	α-SMA	Palliative 5-FU and oxaliplatin	5.5 (high) vs. 15.0 (low) months (*p* = 0.005)	[26]
53 pts with rectal cancer	RT-PCR	High expression of CXCL12 mRNA from microdissection for the stromal region	Neoadjuvant 5-FU and 20-45 cGy radiation	Positive CXCL12: poor overall survival (*p* < 0.01)	[27]
52 pts with rectal cancer	RT-PCR	High expression of CXCL12 and FAP mRNA from microdissection for the stromal region	Neoadjuvant 5-FU and 20-45 cGy radiation	High two genes: poor overall survival (*p* < 0.05)	[28]
141 pts with esophageal cancer	IHC	CXCL1 in CAF	Chemoradiation	High expression: HR 3.347 (*p* = 0.001)	[29]
130 pts with esophageal cancer	IHC	TGF-β in CAF	Chemoradiation	High expression: poor overall survival (*p* = 0.002)	[30]
68 pts with esophageal cancer	IHC	PAI-1 in CAF	Cisplatin	High expression: poor progression-free survival (*p* = 0.0267)	[31]
10 pts with gastric cancer	NanoString	ECM-related gene set in pretreated biopsy tissues	5-FU based palliative chemotherapy	Significantly high in non-response group	[15]

Pts: patients; IHC: immunohistochemistry; CAF: cancer-associated fibroblast; HR: hazard ratio; ECM: extracellular matrix.

**Table 2:** Cancer-associated fibroblast (CAF)-derived factors that can induce treatment resistance in gastrointestinal cancers.

CAF-Derived Factors	Mechanism	Resistant to	Cancer Type	References
Cytokines and chemokines				
IL-6	CXCR7 via STAT3/NF-κB pathway	Cisplatin	ESOC	[70]
	Upregulation of CSC markers	Paclitaxel Carboplatin Radiotherapy	ESOC	[72]
	JAK1/STAT3 signaling pathway	5-Fluorouracil	GC	[15]
CXCL1	MEK/ERK pathway	Radiotherapy	ESOC	[29]
CXCL12/SDF-1	FAK/ERK1/2/AKT signaling pathway Activation of β-catenin and NF-κB	Gemcitabine	PDAC	[77]
	FAK/ERK1/2/AKT signaling pathway Upregulation of IL-6	Gemcitabine	PDAC	[75]
	CXCR4 activation	Radiotherapy	PDAC	[80]
IL-17A	NF-κB pathway	FOLFOX (5-Fluorouracil, oxaliplatin, leucovorin)	CRC	[81]
Growth factors				
TGF-β	FOXO1/TGF-β signaling loop	Cisplatin Taxol Radiotherapy	ESOC	[30]
	-	Cisplatin	ESOC	[85]
IGF	IGF-insulin/IGF1R paracrine signaling axis	Gemcitabine	PDAC	[87]
	IGF1R activation	Radiotherapy	CRC	[89]
HGF	c-MET/FRA1/HEY1 signaling	Cisplatin	HCC	[91]
	MAPK/AKT pathway	EGFR inhibitor (Cetuximab)	CRC	[93]
	-	Radiotherapy	ESOC	[94]
Exosomes				
-	Wnt signaling pathway	5-Fluorouracil Oxaliplatin	CRC	[96]
miR-92a-3p	Inhibition of FBXW7 and MOAP1	5-FU/L-OHP (5-Fluorouracil, oxaliplatin)	CRC	[100]
miR-522	Inhibition of ALOX15 and blocking lipid-ROS production	Cisplatin Paclitaxel	GC	[101]
miR-106b	Targeting TP53INP1	Gemcitabine	PDAC	[102]
lncRNA CCAL	Wnt/β-catenin pathway	Oxaliplatin	CRC	[104]
lncRNA H19	Activation of β-catenin pathway through blocking miR-141	Oxaliplatin	CRC	[105]
-	TGF-β signaling pathway	Radiotherapy	CRC	[105]
Other secreted proteins				
PAI-1	AKT/ERK1/2 signaling pathway	Cisplatin	ESOC	[31]
Perlecan/HSPG2	-	Gemcitabine/Abraxane	PDAC	[109]
Laminin A1	-	Gemcitabine	PDAC	[110]
TIAM1	Stemness through Wnt signaling	5-Fluorouracil Oxaliplatin Irinotecan	CRC	[111]
Hyaluronic acid	Increase interstitial fluid pressure	Gemcitabine	PDAC	[112]
